# Treatment of Varicose Veins by Enucleation

**Published:** 1890-06-14

**Authors:** 


### TREATMENT OF VARICOSE VEINS BY ENUCLEATION.

At the surgical clinic at Rostock, Dr. Boennecken has treated fourteen cases of varicose veins of the lower extremities, complicated with pain, fatigue, cramps, ulcers, eczema, &c., by careful enucleation of the dilated veins, with the best results.

